# The Impact of Environmental Social Media Publications on User Satisfaction with and Trust in Tourism Businesses

**DOI:** 10.3390/ijerph17155417

**Published:** 2020-07-28

**Authors:** Juan Gabriel Martínez-Navalón, Vera Gelashvili, José Ramón Saura

**Affiliations:** Department of Business Economics, Rey Juan Carlos University, 28033 Madrid, Spain; juangabriel.martinez@urjc.es (J.G.M.-N.); vera.gelashvili@urjc.es (V.G.)

**Keywords:** environmental sustainability, satisfaction, trust, social media, SEM

## Abstract

The main aim of the present study was to analyze whether publications related to environmental sustainability in social media directly and positively influence user satisfaction with and trust in tourism businesses. Our second goal was to determine whether the influence of environmental sustainability and satisfaction is moderated by users’ gender. Data collection was performed using a questionnaire. The questionnaire responses were analyzed using the partial least squares-structural equation modeling (PLS-SEM) methodology. The results have shown that there is a positive relationship between environmental sustainability, satisfaction, and trust generated by tourism companies through their publications on social media, and that this relationship is not conditioned by users’ gender. The results of the present study contribute to the literature by bridging the gap in research on tourism enterprises and their strategies regarding social media publications. Our findings also provide important implications related to the content of environmental sustainability strategies and social media communication for tourism companies.

## 1. Introduction

In recent decades, the emergence of social media has provided companies with direct and fast contact with their customers [1]. Nowadays, social media are among the “best possibilities available” means for companies to get in touch with potential customers [2]. By means of social media, companies can announce offers, promote new products and services, get to know about their customers’ preferences, and directly relate their offers [3,4]. Furthermore, several studies have demonstrated that the costs of traditional marketing are considerably higher than social media marketing [5,6,7,8]. 

Other advantages of using social media for companies include transparency, efficiency, and openness [8,9]. Several studies have highlighted the beneficial role of social media for business, showing that appropriate use of social media can help businesses to capture new customers and turn interested people into future customers [6,10,11,12]. 

Furthermore, companies also use social media to promote sustainability [8,13]. In recent years, sustainability, a recently emerging concept, has become a popular word among the general public [14,15]. While the meaning of sustainability varies depending on its context of use, such as social, economic, or ecological [16,17], most interpretations of sustainable development converge in that a company’s policies and actions must be respectful of the environment and be socially equitable if they want to contribute to economic growth [18]. That means that sustainable development should be responsible and include conscientious management of the environment to create a positive impact [19]. Accordingly, since sustainability issues are considered to be strategic topics to ensure companies’ success and operability, companies have been trying to strengthen and grow their sustainability [20,21,22]. In the long run, sustainability is a determining factor for firms’ achieving superior competitive advantage and financial performance [23].

Numerous studies have analyzed the sustainability of Spanish companies operating in different sectors [24,25,26]. Many of these studies have highlighted several important benefits that companies can obtain when they apply sustainability in their management model. However, although several studies emphasized the importance of tourism businesses in Spain [27], relevant research on the sustainability of tourism enterprises in Spain remains scarce. 

At present, due to the rapid increase in the awareness of the population about sustainability issues, environmental sustainability is a key objective of many tourism companies. Accordingly, in order to follow this trend, along with other modifications [27], companies should modify their sustainability policies.

As demonstrated by Reyes-Menendez et al. [28], most tourism companies in Spain are sustainable, and their stakeholders are aware of it. However, the question is whether investing in environmental sustainability and informing stakeholders about it can increase customer satisfaction with and trust in tourism companies. To date, none of the previous studies has addressed this issue. To bridge this gap in the literature, the present study evaluates the influence of social media publications about sustainability on customer satisfaction with and trust in tourism businesses.

The main aim of the present study is to analyze the impact of social media on promoting environmental sustainability. The two research questions addressed in this study are as follows: (1) Can social media publications by tourism businesses about sustainability influence user satisfaction? (2) If so, can user satisfaction affect user trust in tourism businesses that share publications about sustainability in social media? Accordingly, the present study analyzes whether social media publications related to environmental sustainability directly and positively influence satisfaction and, indirectly and positively, user trust in tourism companies. Specific attention is paid to user gender. The analyses are performed from the user perspective, i.e., using a survey. 

The remainder of this paper is structured as follows. Section 2 presents a review of relevant literature on marketing and sustainability in social media. In Section 3, hypotheses are formulated and the sample and methodology are presented. Section 4 reports the results that are discussed further in Section 5. Section 6 concludes.

## 2. Literature Review

### 2.1. Social Media Marketing

In the 21st century, social media have become a new way of communication that enables people to obtain information and express their beliefs and ideas in a new way [29]. Social platforms, such as Facebook, Twitter, YouTube, Instagram, or LinkedIn, have entered people’s daily lives; today, people increasingly use these media for social interaction and information exchange [30,31]. Nowadays, social media are used in different fields like culture, entertainment, education, politics, economy, or business. In the business area, social media offer business owners’ new ways to communicate, collaborate, create [32], or receive feedback from their users [33,34].

One of the areas where social media are actively employed is marketing [2,3,35,36]. Therefore, since the emergence of social media, companies have changed the way they market their products and services [37]. Marketing through social media has become a tool that helps companies keep in touch with their users, promote the products and services, and transform this relationship into sales in the best case [38]. 

Many previous studies have demonstrated that using social media marketing can increase companies’ revenue and efficiency, reduce the expenses, help to stay in touch with potential and actual users, increase the impact on promotional activities, and so on [3,4,39]. Another advantage of social media marketing is its higher cost efficiency as compared to traditional marketing [5,7,40]. Owing to the advantages offered by social media marketing, companies are increasingly trying to use this type of marketing to promote their products and services and attract more customers.

However, marketing through social media also has several limitations. For instance, Maecker et al., [11] found that customers of the companies or specific products that use social media marketing have more service requests. Therefore, if enterprises have more service requests, they should invest more money in this area. Furthermore, Todor [40] reported that disadvantages of social media marketing include the lack of user trust due to the large number of frauds related to virtual promotions, cash-on-delivery systems, copyright issues, and so forth. Accordingly, if companies in areas as diverse as tourism, trade, or culture want to enjoy all the advantages offered by the use of marketing through social media, they should carefully consider potential hazards of this channel of marketing.

### 2.2. Environmental Sustainability in Social Media Marketing of Tourism Industry in Spain

The first official definition of sustainability comes from the United Nations General Assembly Report of the World Commission on Environment and Development. It was initially called “Our common future” and then renamed as the "Brundtland report" prepared by Brundtland et al. [41]. The report defines sustainability as “development that meets the needs of the present without compromising the ability of future generations to meet their own needs” (p.39). The report distinguished three different types of sustainability: environmental, economic, and social. According to Basiago [42], economic sustainability is understood as a production system that meets current consumption levels without compromising future needs, while social sustainability consists of a system of social organization that reduces poverty.

Finally, environmental sustainability requires the maintenance and protection of environmental resources for future generations. Therefore, environmental sustainability refers to different production and engineering processes that support and protect the environmental system [43]. Accordingly, environmental sustainability concerns the preservation and protection of natural resources and the development of alternative sources of energy, as well as maintenance of sustainable consumption levels, development of green products, and promotion of biodegradable products.

Since customers are the main marketing variable for the company, and all properly transferred information determines the success or failure of a business, marketing is an important factor related to sustainability [44]. This has given rise to the emergence of the new concept “sustainable marketing”, which is a combination of marketing and sustainability. 

Fuller [45] defined sustainable marketing as “the process of planning, implementing, and controlling the development, pricing, promotion, and distribution of products in a manner that satisfies the following three criteria: customer needs are met, organizational goals are attained, and the process is compatible with ecosystem” (p. 4). However, despite the centrality of this concept today, only very few previous studies in the area of marketing have analyzed how enterprises communicate sustainability to the public [46].

Numerous studies demonstrated that social media belong to the most popular marketing tools [2,3,36,39]. Therefore, similarities between sustainable marketing and social media can be detected. For instance, Du et al. [13] pointed out that consumers increasingly seem to prefer eco-friendly offers. Accordingly, companies focus on their new sustainable product performance and pass that information on to their users. Using social media to promote the sustainable marketing of new products and services would be beneficial for companies. Several previous studies have investigated the effect of sustainable marketing through social media and its benefits for the companies [13,47]. However, relevant research on Spain, including its tourism sector that significantly contributes to the country’s economic growth, has been scarce [48].

The tourism industry plays an important role in the Spanish economy, generating a considerable dragging effect on other sectors [49]. According to a report prepared by the business association World Travel and Tourism [50], the tourism sector amounted to 14.6% of Spanish GDP.

In addition, travel and tourism created 1 in 5 new jobs over the last five years. Several authors have studied different aspects of the tourism industry, such as accessible tourism for everyone [51], climate change and its associated risks for the tourism industry [52], the importance of evaluating sustainable tourism and tourist destinations [53], cultural tourism and its evolution in the last years [54], or rural tourism facilities [55]. All these studies have emphasized the importance of the tourism industry for the Spanish economy and society. For instance, Rodríguez-Antón et al. [56] reported that, despite the recent emergence of the phenomenon of the so-called collaborative economy, there has been enormous growth and future potential of the collaborative tourism model in the tourism industry. This implies that the tourism industry will continue to grow positively and contribute to the economic development of the country.

## 3. Hypotheses Development

### 3.1. Hypotheses

This section presents the hypotheses to be tested in the present study. Numerous previous studies have shown a strong link between marketing and sustainability and have come to the conclusion that environmental sustainability is a crucial factor for companies to create green products, be competitive in the market, increase profits, maintain employee loyalty, or maintain users trust and satisfaction towards them [57,58,59]. For instance, Walsh and Dodds [59] pointed out that the company’s activities have a significant impact on the environment and society in general. Accordingly, customers become increasingly sensible of the environmental, social, and economic implications of their actions towards reasonable consumption and, therefore, put some pressure on companies to implement environmentally friendly policies. 

In this connection, a study by Pereira-Moliner et al. [60] showed that environmental management practices positively influence financial performance, market success, and the satisfaction of users and employees. However, few studies directly explored the relationship between environmental sustainability and user satisfaction [31]. Based on the considerations discussed above, the following hypothesis can be formulated:

**H1.**  *Social media publications related to environmental sustainability would directly and positively influence user satisfaction*.

According to McMurrian and Matulich [61], companies that generate the trust of their users are more likely to expand and improve their income. Numerous previous studies have analyzed user trust in marketing and management [62,63,64]. For instance, a study on travel agencies demonstrated that the more satisfied is a customer in his/her relationship with the travel agency, the more trust s/he attributed to that agency [65]. Likewise, Kassim and Asiah [66] found that customer satisfaction in e-commerce settings has a positive impact on customer trust. Based on the evidence reviewed above, the following hypothesis can be formulated:

**H2.**  *Satisfaction of social media users with tourism companies directly and positively influences their trust in those companies*.

Furthermore, while previous research on sustainability and satisfaction has demonstrated that more and more consumers support sustainable companies [67,68]. A study by Hou and Elliott [69] showed that compared to men, women are more involved in the search for information, dedicating more time and attention to what they need to know before making their purchase. Similarly, Trivedi and Teichert [70] found gender differences in consumer preferences and purchase intention. Based on this evidence, in the present study, the following hypothesis will be tested:

**H3.**  *The influence of environmental sustainability and satisfaction is mediated by user gender*.

**Figure 1:**
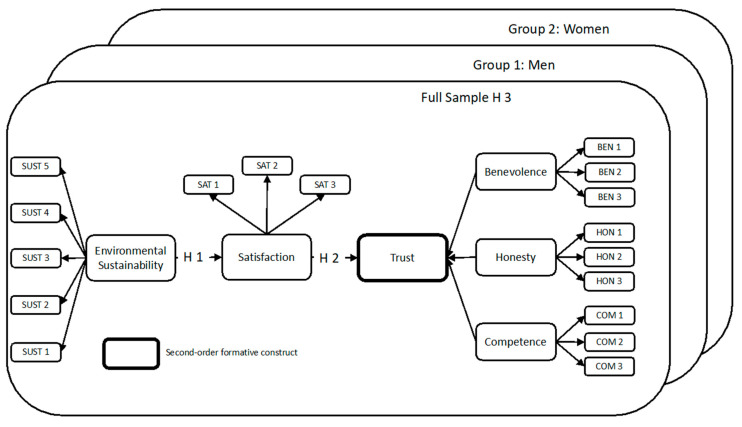
Research model used in the present study.

Based on the hypotheses formulated above, the following model is proposed (see Figure 1).

### 3.2. Sampling and Methodology

This study aims to investigate whether social media publications about environmental sustainability influence user satisfaction and trust in tourism businesses. To this end, an on-line questionnaire was constructed. Data collection was performed in August 2019. The invitation to participate in the questionnaire was disseminated through e-mail and social media (Twitter, Inc., San Francisco, CA, USA) and Facebook (Facebook, Inc., Menlo Park, CA, USA)). Data collection was supported by active tourism companies and hotels in the province of Albacete (Spain); these companies granted the authors access to their customers through social media. The province of Albacete was chosen because this study is part of a series of investigations that aim to measure different variables in Spanish regions that suffer from the risk of depopulation, and where tourism can be one of the industries that can prevent such depopulation. The advantage of selecting this province is the interest shown by the companies to participate in the research since the results will allow them to improve in the future. However, since Spain is a very touristic country, the results of this study might not be generalizable to less touristic countries. 

The questionnaire consisted of two blocks. The first block contained questions about demographic characteristics of the participants; in the second block, the participants were asked to rate a number of items on a 5-point Likert scale (1 = ‘strongly disagree’; 5 = ‘strongly agree’). 

For the data analysis and the validation of the hypotheses, the model for the study of structural equations was used, which has its origin in variances (SEM). This model makes it possible to statistically analyze the predicted relationships by predicting the dependent variables, which makes it possible to calculate and quantify the direct and indirect effects of the variables on each other [71,72]. 

While there are several techniques based on variances, in the present study, the partial least squares (PLS) was used. This technique enables the analysis of composed and factor models, which makes it possible to measure variables and estimate the proposed model [71,73,74].

As argued in many previous studies, the PLS-SEM technique is one of the most complete methods for the analysis of models where relationships between variables are identified and the influence of these among other options is measured [73,75,76,77]. In addition, this technique is used to perform a multi-group analysis [31].

In the present study, a total of 362 questionnaire responses were obtained; however, since some responses were incomplete, only 351 questionnaires were included in the final sample. Therefore, the sample was sufficient for the analysis using the PLS-SEM technique. The use of this technique is also justified in investigating a novel topic on which available literature is scarce and where many different relationships are to be explored. Accordingly, this technique is widely used as an effective tool for exploratory analysis [78]. Using the PLS-SEM technique is also recommended when some of the analyzed variables are composed of dimensions [31], as well as when the proposed model is complex [79]. The PLS-SEM analysis was performed using SmartPLS 3.0 (SmartPLS GmbH, Bönningstedt, Germany), a widely used and reliable software [73,80]. 

## 4. Analysis of Results

### 4.1. Descriptive Analysis

As can be seen in Table 1, gender distribution in the sample was balanced (52% women; 48% men). Most participants (52%) were 31–45 years old, followed by those aged 18–30 years old (19.9%) and 46–55 years old (17.6%). Almost two-thirds of the participants (62.4%) were employees, followed by self-employed (16.8%). In terms of time spent using social media, 37% used social media between 30 and 60 min a day, followed by 28.5% who used them for less than 30 min. 

In terms of the preferred social platform, the respondents were given the possibility to check more than one option. Most participants (88.3%) used Facebook, followed by Instagram (59.8%) and YouTube (54.4%). Snapchat was regularly used by only 5.1% of the participants.

### 4.2. Measurement Model Analysis

The analysis using PLS-SEM unfolded in several steps [73]. First, the validation of the measurement scale was carried out. Second, the structural model analysis was performed. Third, the multi-group analysis investigating whether there was a moderation effect in the predicted relations according to user gender was run.

In the present study, the measurement scale was validated twice: first with the items of the multidimensional variable and, second, with the already grouped dimensions. This led to the establishment of the first- and second-order models [81].

In the first step, we validated the measurement scale twice. For the first-order model, all items of the variables were reflective; thus, the criteria to be tested were individual reliability, composite reliability, convergent validity, and discriminant validity.

In the first section, the items favorably outperformed the cutting indices of the first three criteria used, exceeding the value of 0.707 proposed by Carmines and Zeller [82] for individual reliability, Cronbach alpha of 0.70 in the criterion of Nunnally and Bernstein [83] for composite reliability, and 0.5 of the criterion of Fornell and Larcker [84] which sets the minimum level of average variance extracted (AVE) [75]. In the results, the Cronbach alpha values were very close to 0.9, which can incur possible errors [74]. Therefore, the Dijkstra-Henseler indicator (rho_A) was also analyzed, which gives the study results greater robustness, as it is considered the most reliable measure for the analysis of composite reliability [31,85]. The analysis was positive, as all constructs exceeded 0.7. All of the above indicated that all analyzed constructs were reliable and that they accounted for over 50% of the variance of the corresponding items [73]. Said differently, all constructs exceeded the minimum values of composite reliability and convergent validity (see Table 2).

In the last step of the validation of the first-order measurement scale, discriminant validity was analyzed. This was done by means of two analyses. The first analysis was based on Fornell and Larcker’s [84] method that analyzes the amount of variance captured by a variable from its indicators (AVE); this amount must be greater than the variance that this variable shares with other variables in the model [73].

The second analysis, called heterotrait-monotrait (HTMT), allows for a more rigorous analysis of the discriminatory validity criterion [86]. In this last criterion that can be seen in Table 3, not all items exceeded the threshold. Therefore, the items SAT2, HON2, COM1, and COM2 were eliminated (see Table 2).

Once the first-order measurement scale was validated, a grouping of the items of the multidimensional variable was performed, allowing for the second validation of the second-order model. In this model, the dimensions of the multidimensional variable “trust” had a formative character, which is why other analyses were carried out to validate the second-order scale.

First, all criteria previously made for reflective items were studied; however, in the second-order model, all items were kept (Table 4). Subsequently, the analysis was carried out for the formative variable to rule out collinearity problems. This was done by means of the evaluation of the variance inflation factor (VIF; Hair et al. [73,78]). Then, the magnitude of the variable’s weights [87] and significance [81] was evaluated. Table 4 shows the three dimensions of confidence that fully validate the measurement scale.

### 4.3. Structural Model Analysis

Furthermore, the analysis of the structural model was performed. This analysis aimed to evaluate the predictive capacity of the model and the relations predicted by the hypotheses. Before proceeding to the model analysis, collinearity in the structural model should be ruled out [88]. This is done by means of VIF values of the model which must not exceed 5 [73], followed by the analysis of the algebraic sign, the significance and magnitude of the coefficients: Path coefficients (β), R2 values (variance explained), the size of the effect ƒ2 and the Q2 test (validated cross redundancy) in order to measure the predictive relevance of the model [73,89,90]. 

With regard to the analysis of the predictive power, i.e., the amount of variance (0.75 relevant, 0.50 moderate, and 0.25 weak; see Hair et al. [78]; Henseler et al. [75]) of a variable explained by another predictive variable (determination coefficient R2), the values obtained for the variables were moderate (60%) for trust and weak (36%) for satisfaction.

Subsequently, the effect size of (f2) was analyzed; this value shows the level to which an exogenous variable contributes to explain an endogenous variable. Table 5 reports the results, and the two studied relationships have a large effect size.

As for the cross-validated redundancy indices (Q2), which help to examine the predictive relevance of the theoretical/structural model [87], these indices were greater than zero. Therefore, the model had a satisfactory predictive relevance.

Therefore, the relationships predicted by Hypotheses 1–2 were significant and had high predictive power and a significant influence effect. Therefore, Hypotheses 1 and 2 were supported by the results. The results of Hypothesis 3 are reported in Section 4.4.

### 4.4. Multi-Group Moderating Effect

To perform the analysis of the multi-group moderating effect, the measurement invariance test of the compounds must be carried out [31,81]. Such an analysis, called MICOM, is a prerequisite for carrying out a multi-group study [73,91,92]. In the present study, MICOM unfolded in the following three steps. First, we observed that the two subsamples (men and women) were tested for having the same configurational invariance, i.e., on whether they had exactly the same model. Second, compound invariance was searched for. It was stable, as the scores of a compound using the weights of group 1 did not differ from those created using the weights of group 2. Third, 3a and 3b are divided into two parts: 3a analyses equality of variances and 3b analyses equality of measures. Table 6 shows the results of the MICOM analysis.

The results of the MICOM analysis showed complete invariance, suggesting that both subsamples could be grouped together. Once the satisfactory result of the invariance was obtained, the multi-group analysis was performed using two non-parametric techniques, PLS-MGA and PERMUTATIONS, and one parametric technique, the Parametric Test. Table 7 shows that the results obtained using the different analysis techniques of multi-group moderation (Parametric Test, PLS-MGA, and Permutations).

**Figure 2:**
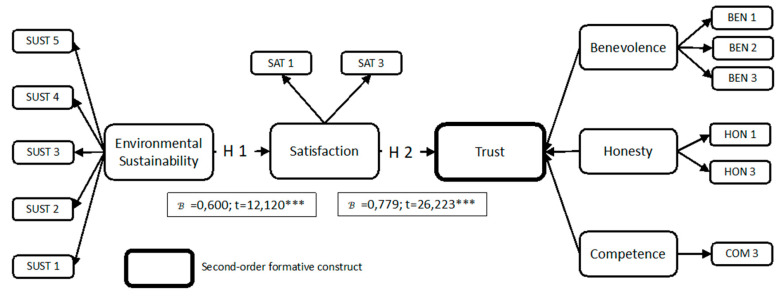
Proposed research model results.

The results of these analyses showed that user gender did not exert a moderating effect on the relationships between satisfaction-trust and environmental sustainability-satisfaction. Therefore, Hypothesis 3 was not supported by the results. Figure 2 shows the model validated for this study.

## 5. Discussion

This study investigated the relationship between social network publications by tourism companies where these companies promote their sustainable strategies and respect for the environment, on the one hand, and user satisfaction and trust, on the other hand. Supporting H1, our results showed that social media publications related to the environment and sustainability influence user satisfaction. This evidence is consistent with the results reported by Reilly and Hynan [17] and suggests that companies should focus on disseminating and preparing communication plans that support sustainable practices and are respectful towards the environment.

According to Azhar and Fauzan [93], user satisfaction is a key factor that plays a fundamental role in the marketing and development strategies of hotel companies, various tourism businesses, or local businesses that travelers explore while traveling. Similarly, to Saura et al. [34], the results of the present study showed that user satisfaction when browsing through social networks must be positive, as it has a real impact on user engagement with the organization. 

Furthermore, supporting H2, our results showed that the satisfaction of social media users with tourism companies directly and positively influences their trust in those companies. This result is consistent with the findings reported by Cori et al. [94], in the political field. Therefore, companies should increase travelers confidence in their sustainable policies, rather than in their messages related to promotions, discounts, or elements that do not add value to the marketing strategy.

In addition, in line with Apperson et al. [95], and Bopp et al. [96], our results showed that, while the strategy of social media publications can improve user trust in tourism businesses, such elements as pollution, society, unsustainable strategies or environmental pollutants in the tourism environment can make tourists feel uncomfortable during their travel.

Based on these findings, it can be concluded that the appropriate content strategy for managers of tourism companies would be to create messages that focus on maintaining values related to the environment [72]. As suggested by Heinonen [97], the messages revealed by companies on social networks can influence user behavior. Another important aspect to consider is how users organize themselves in communities on social networks and interact with the messages that companies launch [98]. These strategies based on social media posts demonstrate user interest in maintaining two-way communication with companies in digital environments [99].

The results of the present study confirm that social media communications should follow a sustainable policy and disseminate relevant environment-related achievements and policies adopted by tourism companies. In this way, users will be able to share the publications with their peers, use those publications for their own purposes, or demonstrate their pride in the fact that they are using the services of an environmentally conscious tourism company to their peers in social network communities. 

Finally, our results showed that gender does not moderate user appreciation of social media publications related to sustainability and the environment. Therefore, our findings do not support [100] as the conclusion about gender influence on user behavior in social networks, particularly in the sustainable tourism sector. In contrast, other authors, such as Palos-Sanchez et al. [101], consider the study of gender as an important factor in drawing robust conclusions.

However, considering that our study is the first attempt to investigate the impact of gender on user appreciation of environment- and sustainability-related publications in social media, this topic deserves further in-depth investigation in future research.

As indicated above, social network publications have been studied from different perspectives [102]. However, there is room for improvement to study the feelings of users when their followers support their publications, adding likes, and sharing content on social networks [103]. 

From this analytical perspective, the study of how users obtain immediate rewards on an emotional level on social media is interesting to set future research objectives in this area. By analyzing these findings, it will be possible to understand how sustainable publications can contribute to creating a positive impact on consumers through social media [104].

In addition, we agree with the results of Tomomi [105], since companies should add environmental supportive content to their communication and marketing plans. A relevant example would be companies’ communicating the content related to supporting sustainable projects and actions, which will both boost their environmental reputation online and make their followers support their content marketing plans by sharing this kind of content.

## 6. Conclusions

In the present study, the analysis unfolded in the following two blocks. In the first block, the proposed model of how environmental sustainability, directly and indirectly, influences social media users’ satisfaction and trust was analyzed. In a second block, we investigated whether the model can be moderated by user gender. Of note, previous studies that analyzed the relationship between sustainability and social media user satisfaction and trust are scarce, which complicated formulating the hypotheses based on available literature. 

The results showed that environmentally sustainable tourism companies generate greater satisfaction among their users. In addition, environmentally sustainable tourism companies also indirectly induced user trust. Therefore, the first two hypotheses tested in this study (H1 and H2) were supported by the results. 

However, our prediction that user gender may moderate the aforementioned relationships was not supported by the results. Therefore, H3 had to be rejected.

### 6.1. Theoretical Implications

As discussed in Section 1, this study aimed to bridge a gap in previous literature by investigating the impact of social media publications by tourism companies on user satisfaction and trust. In doing so, the present study contributes to available knowledge about the role of social media publications in the tourism sector. Furthermore, to the best of our knowledge, the present study is the first to investigate whether user gender plays a role in user evaluation of social media publications related to sustainability and the environment. The results of the present study suggest meaningful directions of further research in this area, including elements such as the organization of users in communities in social networks, the type of interaction according to the message published in the tourism ecosystem, and the concerns and behaviors of users when interacting with this type of content. Our findings also highlight that the content and communication strategies developed by companies in the tourism sector should focus on actions related to environmental sustainability.

### 6.2. Practical Implications

As for the practical implications for the improvement of the management of tourism companies, it should be pointed out that environmental sustainability is a growing asset within the tourism sector. This implies that an organization committed to environmental sustainability and the one that explicitly reveals this message to its customers will substantially improve user satisfaction and the trust of its stakeholders. The implementation of waste separation policies, the use of products with ecological packaging, reduction of energy costs, and other relevant eco-friendly policies will help the company to pollute less and increase its customers’ purchasing possibilities.

Our findings highlight that the communication strategies of tourism companies should explicitly inform their customers about various sustainability policies implemented. This will improve companies’ public profiles, lead to an increase in the number of customers, and thus expand companies’ influence on society.

Finally, since we did not find any gender differences, managers of tourism companies should take into account that in communicating policies, the strategies should not differentiate between men and women, since both groups have the same perceptions of sustainability and environmental issues. Therefore, campaigns should be carried out in the same way for both genders.

### 6.3. Limitations and Future Research

The present study has several limitations. First, the size of the sample was rather limited, particularly given that there are possibilities to get larger samples in the tourism industry. Second, due to the paucity of relevant prior research, it was difficult to ground the formulated hypotheses in past work. Thirdly, due to the novelty of the topic, the theoretical framework that the present study is based on is exploratory. In order to expand the present investigation, further research that would replicate the present study design in other sectors or countries, as well as expand the model by adding other marketing variables, such as commitment, loyalty, economic sustainability, or social sustainability. 

## Figures and Tables

**Table 1:** Characteristics of the study participants (*n* = 351).

Classification Variable	Variable	Frequency	Percentage
Gender	Female	182	52%
	Male	169	48%
Age	18–30	70	19.9%
	31–45	184	52.4%
	46–55	62	17.6%
	56–65	19	5.4%
	>65	16	4.6%
Employment	Student	32	9.1%
	Housewife/man	14	3.9%
	Unemployed	27	7.6%
	Employed	219	62.4%
	Self-Employed	59	16.8%
Minutes devoted to social media per day	<30	100	28.5%
	30–60	130	37%
	60–90	40	11.4%
	90–120	36	10.3%
	>120	45	12.8%
Social media used (multiple responses)	Twitter	126	35.9%
	Facebook	310	88.3%
	Instagram	210	59.8%
	LinkedIn	160	45.6%
	YouTube	191	54.4%
	Snapchat	18	5.1%
	Pinterest	65	18.5%

**Table 2:** Measurement items first order.

Constructs	Items	Correlation Loading	CA	CR	rho_A	AVE
Satisfaction	(SAT1) I am satisfied with the knowledge I get from the social media of the tourism companies I follow.	0.885 ***	0.790	0.904	0.820	0.824
	(SAT3) The social media of the tourism companies I follow meet my expectations.	0.930 ***				
Honesty	(HON1) The tourism companies that I follow on social media keep their promises.	0.933 ***	0.805	0.832	0.832	0.835
	(HON3) The social media of the tourism companies I follow are managed in an ethical and transparent way.	0.894 ***				
Benevolence	(BEN1) The social media of the tourism companies I follow offer beneficial advice and recommendations.	0.884 ***	0.883	0.928	0.885	0.811
	(BEN2) The tourism companies that I follow in social media develop actions taking into account how they will affect their interest groups.	0.871 ***				
	(BEN3) The tourism companies I follow on social media are concerned about the interests and benefits, both present and future, of their stakeholders.	0.956 ***				
Competence	(COM3) The tourism companies that I follow have a knowledge of their users that allows them to adapt to users’ needs.	0.904 ***	1.00	1.00	1.00	1.00
Environmental Sustainability	(SUST1) The tourism companies that I follow on social media have recycling policies.	0.740 ***	0.882	0.914	0.894	0.680
	(SOST2) The social media accounts of the tourism companies that I follow promote positive environmental ethics.	0.869 ***				
	(SUST3) The tourism companies that I follow in social media value and protect the environment.	0.859 ***				
	(SUST4) The social media of the tourism companies I follow publish pollution awareness messages.	0.834 ***				
	(SUST5) The social media of the tourism companies that I follow defend the diversity of nature and promote its value and protection.	0.816 ***				

Note: CA = Cronbach’s alpha; CR = Composite Reliability; rho_A = Dijkstra-Henseler indicator; AVE = Average Variance Extracted; *** *p*-value < 0.001.

**Table 3:** Measurement of the first-order model (discriminant validity).

	Fornell-Larcker Criterion					Heterotrait-Monotrait Ratio (HTMT)				
Constructs	BEN	COM	HON	SAT	SUST	BEN	COM	HON	SAT	SUST
BEN	9.00									
COM	0.693	1.00				0.737				
HON	0.739	0.715	0.914			0.874	0.807			
SAT	0.714	0.644	0.727	0.908		0.850	0.716	0.894		
SUST	0.656	0.538	0.580	0.598	0.825	0.728	0.561	0.669	0.683	

Note: BEN = Benevolence; COM = Competence; HON = Honesty; SAT = Satisfaction; SUST = Environmental.

**Table 4:** Second-order measurement model of the formative construct.

Construct	Dimensions	Correlation (Weights)	CA	CR	AVE	VIF
TRUST	Honesty (HON)	0.483 ***	n/a	n/a	n/a	2.669
	Benevolence (BEN)	0.432 ***				2.511
	Competence (COM)	0.184 **				2.328

Note: CA = Cronbach’s alpha; CR = Composite Reliability; AVE = Average Variance Extracted; VIF = Variance inflation factor; ** *p*-value < 0.01, *** *p*-value < 0.001; n/a = Not applicable.

**Table 5:** Results for Hypotheses 1–2.

	Path Coeff (β)	Statistics t (β/STDEV)	f2	Confidence Interval	
				5.0%	95.0%
H1. Environmental sustainability → Satisfaction	0.600 ***	12.227	0.563	0.723	0.823
H2. Satisfaction → Trust	0.779 ***	25.992	1.547	0.512	0.675

R2: Trust = 0.607; Satisfaction = 0.360; Adjusted R2: Trust = 0.606; Satisfaction = 0.358; Q2: Trust = 0.460; Satisfaction = 0.275. Students in single queue *** *p* < 0.001.

**Table 6:** Results of invariance measurement testing using permutation.

Constructs	Invariance	Composition Invariance		Partial Invariance	Equal Mean Assessment			Equal Variance Assessment			Full Measurement Invariance Established
		C = 1	Confidence Interval		Differences	Confidence Interval	Equal	Differences	Confidence Interval	Equal	
TRUST	Yes	0.999	(0.975/1.000)	Yes	−0.043	(−0.219/0.221)	Yes	0.046	(−0.482/0.469)	Yes	Yes
SAT	Yes	1.000	(0.999/1.000)	Yes	−0.144	(−0.225/0.219)	Yes	−0.074	(−0.393/0.390)	Yes	Yes
SUST	Yes	1.000	(0.997/1.000)	Yes	−0.010	(−0.217/0.221)	Yes	−0.197	(−0.403/0.400)	Yes	Yes

Note: SAT = Satisfaction; SUST = Environmental Sustainability.

**Table 7:** Result for Hypothesis 3.

	Path Coefficient			Confidence Interval (2.5%; 97.5%)	*p*-Value Difference			
Relationship	Men	Women	Difference		PLS-MGA	Permutation Test	Parametric Test	Supported
SAT→TRUST	0.752	0.806	−0.054	(−0.117; 0.118)	0.821	0.360	0.185	NO/NO/NO
SUST→SAT	0.593	0.609	−0.016	(−0.194; 0.192)	0.569	0.872	0.435	NO/NO/NO

Note: SAT = Satisfaction; SUST = Environmental Sustainability.
